# Doctors', Patients' and Physician Associates' Perceptions of the Physician Associate Role in the Emergency Department

**DOI:** 10.1111/hex.14135

**Published:** 2024-07-10

**Authors:** Nicole M. A. King, Suzannah Helps, Yee Gan Ong, Sandra Walker

**Affiliations:** ^1^ Faculty of Science and Health, School of Health and Care Professions University of Portsmouth Portsmouth UK; ^2^ Emergency Department/Acute Medicine Epsom & St Helier NHS Trust, St Helier Hospital Carshalton UK

**Keywords:** emergency department, healthcare, patient engagement in research, perceptions, physician associate

## Abstract

**Introduction:**

The Emergency Department (ED) has seen increased patient attendance and difficulty meeting demands. New healthcare professions such as Physician Associates (PAs) are being utilised to complement the existing medical workforce. Despite the growth of their professions in the United Kingdom, little evidence is available about the perceptions of their roles.

**Objective:**

This study aims to provide evidence of doctors', PAs' and patients' perceptions of the PA role in the UK ED.

**Methods:**

A mixed methods approach consisted of the following:

1. An online exploratory survey of ED doctors at one English ED over 1 month (February–March 2022).
2. Post consultation semi‐structured patient questionnaires over 2 weeks (April 2022).
3. Semi‐structured virtual interviews with ED consultants across the four regions of the United Kingdom (3 months in 2022).
4. Semi‐structured virtual interviews with ED PAs across the four regions of the United Kingdom (3 months in 2022).

The analysis methods that were used included frequency counts and percentages from closed questions, and hybrid thematic analysis of free text and interview transcripts.

**Results:**

Four ED consultants and four ED PAs across the United Kingdom were interviewed. Twenty‐eight ED doctors participated in the online survey. Fifty‐seven patients completed the post consultation questionnaire. Four main themes (PAs being fit for purpose; patient recognition of PAs, PAs providing continuity of care, and future PAs and regulation) were deduced as per the General Medical Council, Good Medical Practice domains (knowledge, skills and development; patients, partnership and communication; colleagues, culture and safety; and trust and professionalism). Other subthemes were induced via hybrid thematic analysis. In this study, doctors and patients had mixed comments about the role of PAs. Most of them were positive as doctor participants perceived PAs to be knowledgeable, highly skilled, with mostly good communication skills, team players, providing continuity of care and overall being fit for purpose. However, some doctor participants commented negatively about PAs for providing little quality healthcare and being inexperienced. There was a desire for career progression among the PA participants and a need to work to their full potential. Although the clinicians of this study displayed a clear understanding of the PA role in the ED, a high frequency of surveyed patients mistook PAs for doctors. It was suggested that future PAs could complete a postqualification programme in emergency medicine, combine roles, be paid on an alternative scale and be formally regulated.

**Conclusion:**

In this study, mixed views were expressed by ED consultants, ED junior doctors and patients regarding the role of the PA in the ED. Stakeholders can use the information presented to develop a better understanding of the perceptions of the PA role within the UK ED.

**Patient or Public Contribution:**

The Patient and Public Involvement and Engagement (PPIE) group, led by Healthwatch, made significant contributions to the study's design by providing valuable feedback on the information sheets and consent forms utilised. The patients' responses helped guide the study's direction and shape its future work. As part of the dissemination activities, the study findings was shared with both the PPIE team and Healthwatch media production team.

**Abbreviations**
ACP: Advanced Clinical Practitioner
AfC: Agenda for Change
BMA: British Medical Association
CCT: Completion of Training Certificate
CDU: Clinical Decisions Unit
CPD: Continual Professional Development
CT: computerised tomography
ECG: electrocardiograph
ED: Emergency Department
EPIC: Emergency Physician in Charge
FPA: Faculty of Physician Associates
FY1: Foundation year 1 doctor in training
FY2: Foundation year 2 doctor in training
GMC: General Medical Council
GMP: Good Medical Practice
GP: general practitioner
Int: virtual interview
ITU: Intensive Therapy Unit
MDT: multidisciplinary team
NHS: National Health Service
OLS: online survey
PA: Physician Associate
PCQ: patient consultation questionnaire
PPIE: Patient and Public Involvement and Engagement
RCEM: Royal College of Emergency Medicine
REC: Research Ethics Committee
SHO: senior house officer
UK: United Kingdom
USA: United States of America

## 1 Introduction

The concept of healthcare delivery has evolved to embrace a multidisciplinary team (MDT)‐based approach [1]. While the idea of team‐based care was first mentioned in the 1920s, the focus was primarily on doctors [2]. Today, patient care is a collaborative effort involving multiple healthcare professionals, and the practice of medicine is no longer the exclusive domain of doctors [3]. In the United Kingdom, the National Health Service (NHS) is battling with several challenges because of a rapidly ageing population, insufficient funding and a shortage of healthcare workers [4]. The shortage of doctors has reached alarming levels, with 10,000 doctors ‘relinquishing’ their licences to practice in 2021, representing a loss of one‐tenth of the total NHS doctor workforce [5]. Effective teamwork is crucial to improving patient experiences and outcomes as well as providing support for healthcare professionals' well‐being [6]. Physician associates (PAs), formerly known as physician assistants in the United Kingdom, are a relatively new health profession that brought into the healthcare system in 2003 to improve and expand the existing medical workforce in response to increasing patient demands [7]. There has been a rapid expansion of the role with currently > 4000 qualified PAs registered on the Physician Associate Managed Voluntary Register [8] and an expected annual rise of 1300 qualified PAs via 36 UK universities from 2023/2024 to establish a UK PA workforce of 10,000 PAs by 2036/37 as per the NHS Long Term Plan [9]. As part of the plan, there is also an aim to increase the doctor workforce to 60,000–74,000. This role is long established in places such as the United States, where it has been in existence since the 1960s [10]. The notion behind the introduction of PAs in the United States was a solution to a shortage of generalist physicians [11]. The first class of PAs in the United States consisted of four Navy hospital corpsmen who received considerable medical training during their military training [12]. In the United Kingdom, PAs, at a minimum, have an undergraduate degree, usually health or life science‐related, before embarking on a 2‐year intensive medically oriented postgraduate qualification and national certification [13]. The role of a PA involves conducting and recording patient histories and physical examinations. They may also need to order (blood tests and nonionising radiation) and perform diagnostic or therapeutic procedures depending on their training and experience, with the appropriate level of supervision. They are also responsible for formulating and executing management plans, recommending medication, instructing patients, encouraging health promotion and referring patients to other healthcare professionals when necessary [13]. The UK government is in the process of formally regulating PAs with the General Medical Council (GMC). The regulation is expected to be completed by the end of 2024 [14]. Currently, PAs are not allowed to order ionising radiation, including X‐rays or computerised tomography (CT) scans [15]. Although with experience, PAs can display a level of autonomy, they will always remain dependent practitioners with supervision directly or indirectly by a senior doctor (consultant or general practitioner [GP]) [13]. PAs are, however, professionally responsible for their own actions in practice; so are advised to have their own separate indemnity in addition to the institutions (NHS) [16].

The emergency department (ED) has become increasingly busy with rising numbers of patient attendances [17]. With the additional pressure of medical staff shortages, workforce planning has seen an interest in employing other medical professionals such as PAs to complement the existing ED medical workforce [18]. In the United Kingdom, according to the 2022 PA census, 12% (385) of the 3208 PAs who completed the census noted that they worked in the ED, which is a 3% increase in the proportion of PAs working in the ED from the previous year [19]. In the United States, by the end of 2020, 13,219 certified PAs self‐identified as working in the ED [20]. Our recent systematic scoping review found that there is limited formal evidence on how patients, doctors and PAs perceive the role of PAs in the UK ED and the activities that PAs carry out in this setting [21]. Through exploring the patient, doctors and PA perception of the PA role in the UK ED, this study aimed to bridge gaps in the literature to provide better insights for the public, managers, clinicians and commissioners who may be interested in employing PAs in emergency services.

## 2 Materials and Methods

This study was part of a wider study and was mixed‐methods in design, conducted in multiple centres and comprised the following:

(a) An online explorative survey conducted from 2 February 2022 to 2 March 2022 (one English ED).
(b) Patient post consultation questionnaires conducted from 11 April 2022 to 24 April 2022 (one English ED).
(c) Semi‐structured virtual interviews with ED PAs conducted 11 February 2022 to 17 May 2022 (four EDs across four regions of the United Kingdom).
(d) Semi‐structured virtual interviews and ED consultants conducted 11 February 2022 to 17 May 2022 (four EDs across four regions of the United Kingdom).

**Table 1:** Differing titles of doctors according to the levels between the United Kingdom and the United States.

UK	USA
Consultant	Attending
Senior registrar or middle grade	Chief resident
SHO to registrar level 3	Resident
FY1 and FY2	Intern

*Note:* Currently, in the United Kingdom, all doctors below the consultant level are classed as junior doctors. PAs are not yet formally categorised but, in this study, a junior PA = in their first‐year postqualification versus senior PA = more experienced. Senior registrar is level 4 or 4‐year postregistrar level. (ED consultants in the United Kingdom are the most senior level of doctors in the ED who hold a completion of training certificate [CCT]. A UK consultant is equivalent to an ‘attending doctor’ in the United States. Table 1 outlines the differences in titles of the different levels of doctors in the United Kingdom compared to the United States.)

Abbreviations: FY1, foundation year 1 postqualification; FY2, foundation year 2 postqualification; SHO, senior house officer (≥ 3 years postqualification); UK, United Kingdom; USA, United States of America.

### 2.1 Setting

The study was conducted in five university district general hospitals that had Type 1 EDs with 24‐h consultant‐led services and full resuscitation facilities. These hospitals generally dealt with most accidents and emergencies, and two had trauma units. Out of the five hospitals, two were located across England, one in Wales, one in Northern Ireland and the other in Scotland. All the hospitals had between 240 and 629 beds outside of the ED and yearly adult ED attendances of between 50,000 and 70,000 patients.

The study population consisted of ED consultants, ED junior doctors, ED PAs and patients seen by ED PAs.

### 2.2 Ethics, Consent and Permissions

Ethical and research governance approval for this study was obtained from the NHS London Bridge Research Ethics Committee (REC Reference: 221/PR/0802). Informed consent was obtained from the patients, doctors and PAs who took part in the study.

### 2.3 Reflexivity Statement

The study team comprised a main researcher who, at the time, had 8.5 years of working experience as an emergency medicine PA at the site participating in the online survey and patient post consultation questionnaire. The main researcher conducted the virtual interviews, and participants were encouraged to be open and honest in their answers.

### 2.4 Recruitment Strategy and Data Collection

#### 2.4.1 Online Exploratory Survey

**Figure 1:**
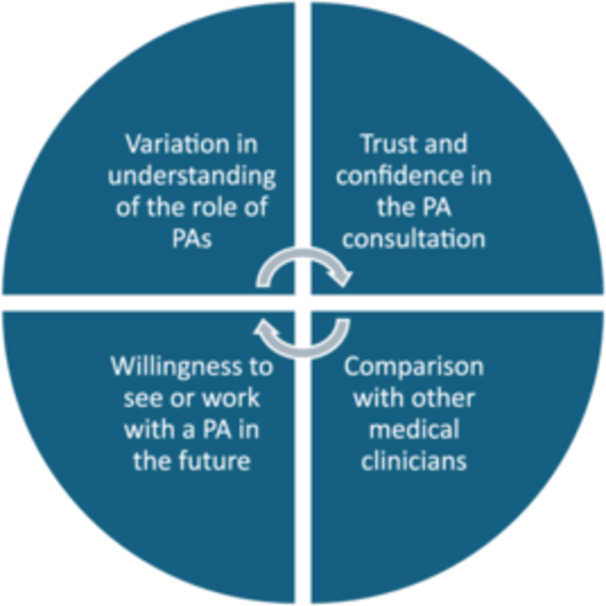
Four standard college satisfaction themes set by the General Medical Council (GMC). PA, physician associate.

This aspect of the study was conducted at one of the English EDs. The SurveyMonkey link was sent to the clinical director's secretary via secure NHS email, to disseminate to all of the current ED doctors (*n* = 35) at the hospital. The link included information regarding the study and a section for consent. All doctors from the ED who had worked with PAs for at least 2 months in an ED setting were included (progression to subsequent questions was aborted for those who had only worked with ED PAs for less than 2 months). The survey was based on the standard GMC colleague satisfaction surveys with a section for free text (Figure 1). The survey consisted of 12 questions, 11 of which are closed in nature. The average completion time was 10 min. The responses were fully anonymised (nonlinked). To increase the response rate, reminders were sent twice via email. The link was then disabled a month after it was initially sent out for completion. The survey was set up to allow only one response per device, to prevent duplicate completions from a single clinician.

#### 2.4.2 Patient Post Consultation Questionnaire

Purposive sampling was used as a methodology to recruit patients. All patients being seen by PAs in one English ED were invited to participate in the study after they had initially been seen by the PA and were waiting for their results or discharge. Patients received an information sheet from the PA or researcher and had time to consider the study. If they agreed to participate and were deemed to have the capacity, informed consent was obtained, and they were provided with the questionnaire (five questions, three open and two closed; Supporting Information 1) in an envelope. After completing the questionnaire, the patient handed it to the reception team in an envelope to be placed in a secure box at the end of their consultation. Patients’ relatives, carers or anyone accompanying them were allowed to stay with the patient if desired. The questionnaire was composed of five, both open and closed, questions exploring the patient's satisfaction (patient understanding of the PA role, ratings of the encounters and willingness to be seen by a PA in the future) of their consultation with the PA. The duration of the questionnaire was around 5 min.

The inclusion criteria for the post consultation questionnaire are outlined as follows:

- Any sex
- 18 years old and over
- Attending the adult ED during the study period
- Being seen by a PA
- Able to provide informed consent to participate

##### 2.4.2.1 Minimising Bias

The questionnaires were completed in the absence of the PA. The questionnaires had no request for patient‐identifiable or traceable (date or time) information. Completed consent forms and questionnaires were kept separate to protect patients' anonymity. Patient feedback is regularly collected within the ED, so it is unlikely that the PA's performance would have been influenced by them being aware that the study was assessing their patient's satisfaction with their consultation. However, to minimise bias, the PA was blinded to the specific questions being asked on the questionnaire.

#### 2.4.3 Virtual Structured Interviews (PAs and ED Consultants)

An advertisement containing the main researcher's contact details was sent to the clinical director (identified via the hospital's websites) of each of the region's EDs via secure webmail to disseminate to the ED PAs and ED consultants. The information sheet and consent forms were sent via secure webmail to all those PAs and consultants expressing interest in the study. Enough time was allowed to obtain informed consent electronically via NHS webmail. An ED PA and ED consultant from each hospital recruited in the study were invited to participate in a semi‐structured virtual interview via Microsoft Teams. The separate interviews were generally about 10–15 min in duration and consisted of seven questions (Tables 2 and 3), which were also based on the standard GMC colleague satisfaction surveys (Figure 1).

**Table 2:** Virtual interview questions for the Emergency Department (ED) physician associates (PA).

1. How long have you been a PA?
2. Was the ED your first job after qualifying? If no, which speciality did you do before?
3. How has your qualifications equipped you to perform your role in the ED?
4. What are the positive aspects of your role in the ED?
5. What are the negative aspects of your role in the ED?
6. What would help improve the development of your role in the ED?
7. Where do you see the future role of the PA in the ED?

**Table 3:** Virtual interview questions for the Emergency Department (ED) consultants.

1. **H**ow long have you worked with PAs in the ED setting?
2. What is your overall impression of their capabilities?
3. How confident are you about their ability to clerk patients in comparison to other medical clinicians?
4. Omitting patient‐identifying information could you describe a recent consultation experience with a PA?
5. What are the positive aspects of their role?
6. What are the negative aspects of their role?
7. How do you see the role of the PA in the future?

*Note:* Clerk = to perform a thorough medical examination; record the findings in the medical notes; and develop a comprehensive diagnosis, problem list and care plan.

A follow‐up reminder email was sent after 5 days to improve uptake. Once the signed consent form was received, the main researcher scheduled time slots to conduct the individual virtual interviews via Microsoft Teams, which were recorded. Within 10 days of the recording, the main researcher generated anonymised transcripts via transcribing the responses verbatim. Once transcribed, the recordings were deleted. The transcripts were not returned to the participants for further comments.

### 2.5 Data Analysis

#### 2.5.1 Data Preprocessing of the Virtual Interviews

The main researcher preprocessed the interview transcripts by (a) carefully reading them line by line; (b) removing the interviewers' text, including any transcribed questions or statements; and (c) organising the remaining unstructured text data into a list according to the corresponding question numbers.

#### 2.5.2 Categorical Data

The closed questions on the staff online survey, patient post consultation questionnaire and virtual interviews were classed as categorical data and analysed via Microsoft Excel (frequencies and percentages).

#### 2.5.3 Hybrid Thematic Analysis

**Figure 2:**
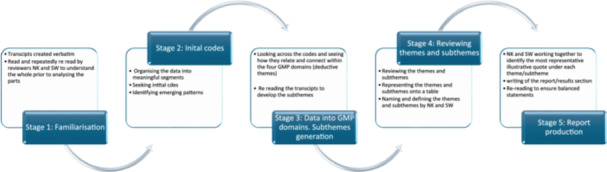
Hybrid thematic analysis process. Adapted [23]. GMC, General Medical Council; GMP, Good Medical Practice.

A variation of traditional thematic analysis by Braun and Clarke using both inductive and deductive themes, known as hybrid thematic analysis, was used [22, 23]. The approach was informed by Schutz's theory of social phenomenology [24], whereas the data‐driven inductive approach was underpinned by Boyatiz and the deductive a priori themes approach by Crabtree and Miller [25]. Free text from the surveys and virtual interview transcripts were analysed via deductive (structured around the GMC's Good Medical Practice domains) and inductive (emerging subthemes) hybrid thematic analysis [26]. The two reviewers read and repeatedly reread the preprocessed transcripts of the verbatim text line by line and became familiar with the text by immersing themselves into the data. The themes and subthemes were generated, inputted onto a Microsoft Excel Spreadsheet, scrutinised and discussed between the reviewers (Figure 2). Triangulation was applied to the virtual interviews from PAs and consultants that were collated under the induced or deduced themes as well as the doctor online survey results and patient responses from the post consultation questionnaire [27].

##### 2.5.3.1 Quality Assurance

Quality was assured via the use of hybrid thematic analysis that is said to add more robustness to the data [23]. Triangulation was also used where one data set informs another such as the virtual interviews from the consultants, PAs, doctor online survey responses and patient questionnaires [27]. During the virtual interview, respondent validation was ensured by repeating the respondent's responses [28]. To ensure a well‐rounded evaluation, we read each transcript independently and then reviewed them together, taking into account both perspectives. This approach allowed us to avoid any initial biases and provided a fair and constructive assessment. Furthermore, we took great care to thoroughly review the transcripts on multiple occasions, ensuring that we were able to clearly understand and interpret all of the information presented. Because of the researcher's insider status, a reflexive lens on all aspects of the research was applied. Quantification of the views from a wide range of perspectives enabled interpretation of the findings and any contradictions to ensure a single response is not presented as if it represents the sole perspective of the ED PA role. To ensure well‐rounded conclusions, we sought feedback from an acute medicine consultant and an associate professor in Health and Social Care Evidence & Evaluation during the manuscript review process. The study was reported as per the consolidated criteria for reporting qualitative research (COREQ; Supporting Information 2).

## 3 Results

### 3.1 Categorical Data

#### 3.1.1 Doctor's Perception (Online Survey)

The survey was sent to 35 doctors (consultants, registrars, middle grades, senior house officers [SHO] and foundation doctors) working in the ED during the study period. Twenty‐eight doctors consented and completed the online survey via Survey Monkey (80% completion rate). The majority (25%) of the participants were consultants (*n* = 7), registrars (*n* = 6), senior house officers (SHO; *n* = 6), middle grades (*n* = 2), foundation year 2 (FY2; *n* = 5) and FY1s (*n* = 2). Fifty percent (*n *= 14) of the doctors worked with PAs for over 1 year: 21.4% (*n* = 6) 6 months to a year and 28.6% (*n* = 8) for 2–6 months. The results of the categorical data are listed in Table 4.

**Table 4:** Demographics and background information concerning the participants (*n* = 28).

Professional role	*n* (%)	Number of years worked with ED PAs	*n* (%)
Consultant	7 (25)	Less than 2 months	0
Registrar	6 (21.4)	2–6 months	8 (28.6)
Middle grade	2 (7.1)	6 months to 1 year	6 (21.4)
Senior house officer	6 (21.4)	Over 1 year	14 (50)
Foundation Year 1 doctor in training	2 (7.1)		
Foundation Year 2 doctor in training	5 (17.9)		

**Questions relating to the role of the physician associate (PA)**	**Poor**	**Satisfactory**	**Good**	**Excellent**	**Unsure**
1. Rating of PAs' ability to see patients with undifferentiated diagnoses	2 (7.1)	1 (3.6)	13 (46.4)	12 (42.9)	0
2. Rating of the PAs' ability to take medical histories	1 (3.6)	2 (7.1)	7 (25)	18 (64.3)	0
3. Rating of PAs' ability to carry out clinical examinations	2 (7.1)	1 (3.6)	10 (35.7)	15 (53.6)	0
4. Rating of PAs' ability to formulate differential diagnoses and management plans	2 (7.1)	2 (7.1)	11 (39.3)	13 (46.4)	0
5. PAs' ability to develop and deliver appropriate treatment and management plans for the patients in the ED	2 (7.1)	3 (10.7)	11 (39.3)	12 (42.9)	0
6. PAs' ability to perform diagnostic and therapeutic procedures	2 (7.1)	4 (14.3)	12 (42.9)	7 (25)	3 (10.7)
7. PAs' ability to interpret diagnostic studies	2 (7.1)	2 (7.1)	15 (53.6)	9 (32.1)	0
8. PAs' ability to provide health promotion and disease prevention advice for patients in the ED	2 (7.1)	1 (3.6)	8 (28.6)	17 (60.7)	0

Abbreviation: ED, Emergency Department.

#### 3.1.2 Patient Perception (Post Consultation Questionnaire)

Fifty‐seven patients gave informed consent and completed the post‐PA consultation questionnaire, which accounted for 100% of the patients who were approached and had the capacity to provide informed consent. The results of the categorical data are listed in Table 5. Of the respondents, 46/57 (81%) had no negative comments about their experience with the PA. Negative comments received were centred around PA role purpose, PA's lack of confidence and increased wait times. These findings were incorporated into the overall hybrid thematic analysis.

The only negative aspect of my experience was the waiting time to be seen was too long.

[Patient 47, PCQ]

I couldn't really see the point of it.

[Patient 18, PCQ]

**Table 5:** Patient responses to the categorical questions on the post consultation questionnaire.

**Category**	**Respondents: *n* (%)**
How would you rate the care you received from the physician associate? (Please tick your response)	
Poor	0
Fair	0
Good	8 (14.0%)
Excellent	48 (84.2%)
Unanswered	1 (1.8%)
Would you be keen to be seen by a physician associate in any future attendances to the Emergency Department (Please tick your response)	
Yes	55 (96.5%)
No	0
Unanswered (unsure written by the patient)	2 (3.51%)

#### 3.1.3 Virtual Interviews (Consultants and PAs)

One hundred percent of the set purposive sample of consultants (*n* = 4) and PAs (*n* = 4) in the four regions were recruited and gave informed consent. The four consultants (three males and one female) interviewed worked with PAs between 4 and 15 years (average 8.5 years). The four PAs (three males and one female) interviewed had been qualified between 3 and 10 years (average 7.1 years). Three of the PAs interviewed had worked in the ED since qualifying, whereas one PA came into the ED after an internship with Trauma & Orthopaedics and combined the ED role with a teaching post.

#### 3.1.4 Triangulated Hybrid Analysis Outcomes

##### 3.1.4.1 Key Themes

The following four themes were identified from the thematic analysis: ‘PAs being fit for purpose’; ‘patient recognition of PAs’; ‘PAs providing continuity of care’; and ‘the future PA and registration’. These themes were then aligned with the four GMC GMP domains [26] discuss the results, which are as follows:

1. Knowledge, Skills and Development,
2. Patients, Partnership and Communication,
3. Colleagues, Culture and Safety,
4. Trust and Professionalism.

**Figure 3:**
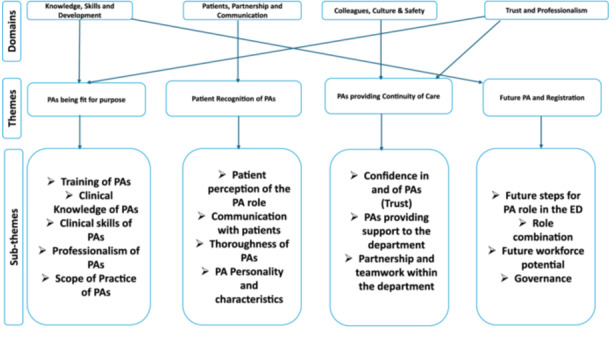
Coding tree: four General Medical Council, Good Medical Practice domains; four key themes and associated subthemes generated by thematic analysis [23]. ED, Emergency Department; PA, physician associate.

The four domains, key themes and their associated induced subthemes are represented schematically in Figure 3. The themes and their associated subthemes are discussed below with some illustrative quotes. Further illustrative quotes can be found in the Supporting Information 3.

## 4 Theme 1: PAs Being Fit for Purpose

### 4.1 Training of PAs

Impressions of PA training varied across regions, with seven consultant participants noting differing competence levels among PAs. Three consultant participants viewed PAs as well‐trained and skilled, particularly for the ED because of their medical model training.

Overall impressions of their [PAs] capabilities. That's a difficult question to ask because that is like asking what is the overall capability of a doctor is because it depends on where they are and what they have been trained in doing. PAs in general have massive potential. But there is always a bit of a fight going on about the PA role against the ACP role. And I will be honest, I feel the PA role is more preferable due to the PA training model. Yes it is a shorter choice, but it is a kind of medical model of training.

[Consultant 1; Int, 15‐year experience with PAs]

A consultant interviewed, mentioned the intensive training of PAs linking to their clinical acumen, making them safe, effective and dependable members of healthcare teams.

Physician Associates are an invaluable resource and have rapidly become core members of the team. Their skill, knowledge and commitment as a group to patient care and the smooth running of the department is unparalleled. They bring a particularly sharp clinical acumen honed by their intensive training, which makes them safe, effective and dependable practitioners.

[Consultant 8; OLS, > 1‐year experience with PAs]

However, a consultant and PA interviewed identified a gap in ED‐specific knowledge for new graduates. Two PAs perceived their training provided a foundation for ED work. The national exam as quoted by a PA and consultant participant and aspects such as Intermediate Life Support certification as quoted by a PA were seen as beneficial for work in the ED.

I think the background learning from the PA course gave me the underlying anatomy, physiology and pathology. The training received on the PA course gave me the background baseline in order in order to kind of learn more of an apprentice in my ED role.

[PA 4; Int, 10‐year experience]

The generalist training received, and the curriculum followed allows for delivering 1st contact medical care at the point of graduation …. I think the training and national examinations help to demonstrate that [generalist] ability, but equally when you start working you know you are working under supervision either directly or indirectly with the senior doctor to you ensure you are managing your patients appropriately and working in your scope of practice.

A consultant noted while PAs can perform basic tasks postqualification, they need support for more complex duties. Despite this, most doctors surveyed rated PAs’ abilities to formulate diagnoses and management plans as either excellent (46.4%) or good (39.3%; Table 4).

… like all clinicians, whether doctor, ACP or PA, the skills that each person has can vary and the level of competence can vary widely. All PAs arrive in the ED being able to take a history and a basic examination, and with time and much clinical supervision and support they develop as individuals in their level of competency of the more complex skills of diagnostics, management, and investigation.

[Consultant 5; OLS, > 1‐year experience with PAs]

### 4.2 Clinical Knowledge of PAs

Nine patients mentioned the knowledge of the PAs. Two patients found the PAs to be very knowledgeable; two patients noted their good understanding of the patients' conditions; one patient found the PA to be informative; and three patients felt reassured by the PAs' knowledge and appropriate management.

I felt I was listened to by someone knowledgeable.

[Patient 10, PCQ]

Very caring, answered all of my questions, made me feel safe and reassured. Very knowledgeable.

[Patient 13, PCQ]

Two PAs interviewed and two patients mentioned the generalist knowledge of PAs.

We *[PAs]* see a broad range of presentations, so we see children right up to the elderly and frail.

[PA 3; Int, 8.5‐year experience]

They have a broad range of knowledge across adults and children. It sounds like they fall between doctors and nurses. They handle patients, but they do have to consult with specialists.

[Patient 36, PCQ]

A PA and consultant interviewed stated that more senior PAs share their knowledge when teaching or guiding other members of staff in the department.

I don't like the word Clerk but would rather use the words assess and treat the patient appropriately and have the knowledge to be able to make sensible decisions, they can all do that. I would say some of our more senior PAs are probably able to support our junior doctors in such a way that they can guide them. We don't actually ask them to do that, but they actually have that experience and knowledge which only comes with a time.

[Consultant 1; Int, 15‐year experience with PAs]

A consultant interviewed regarded PA's knowledge in the ED to be of a high standard bringing focus and competency to the department. The six yearly revalidation examination as a method of keeping up the PAs' generalist knowledge was mentioned by a PA and consultant.

An SHO surveyed and two consultants interviewed regarded PAs on the whole, as having a good knowledge of emergency medicine that enables most of the PAs to have confidence to work within the team to manage patients appropriately.

To a great extent they gain skill experience easily, almost all of them have confidence of medical knowledge and utilise it to provide above expectations care.

[SHO 6, OLS, 2‐ to 6‐month experience with PAs]

However, a doctor participant surveyed noted that PAs have as expected less physiological, pharmacological and biochemistry knowledge.

…. As might be expected slightly less physiological, pharmacological, and biochemistry knowledge which sometimes makes a difference, but less in ED than medicine.

[SHO doctor 4, OLS, 6‐month to 1‐year experience with PAs]

Two doctor participants from the online survey regarded PAs as being inexperienced and lacking quality.

A limited role with inexperienced staff *[PA]* that doesn't add quality to patient care. It is just another pair of hands to see patients.

[FY2 doctor 5, OLS, 2‐ to 6‐month experience with PAs]

### 4.3 Clinical Skills of PAs

Impressions of PA training varied across regions, with six consultant participants who noted different competence levels among PAs. Two consultants viewed PAs as well‐trained and skilled, particularly for the ED. Four consultants viewed PAs as well‐suited for the ED, noting their ability to clerk patients, conduct examinations and request appropriate investigations. The online survey reflected this, with 64.3% of doctors rating PAs' ability to take medical histories as excellent and 25% as good, 53.6% rated their clinical examinations as excellent and 35% as good. A consultant interviewed also highlighted PAs' versatility in handling various cases and creating management plans.

We discuss virtually all of our cases with our PAs …. They see the full spectrum of ED cases and I will leave them to carry out a management plan and review accordingly.

[Consultant 2, Int, 5‐year experience working with PAs]

… there was a patient who came up who had been sent up by the GP with personality change, delusional and paranoid behavior with no other psychotic symptoms. Our PA went to take a history and did a very thorough history and unearthed that about 5 weeks before there had been a head injury that went unreported and added in a possibility of their symptoms being related to an organic cause as opposed to a psychiatric cause. One of the drawbacks of the PAs is that they can't prescribe or request radiology. But based on what I was told I thought it was perfectly reasonable to go ahead and get a CT scan based on what the PAs assessment was, and they [The patient] had a subdural. So that is an example of a PA who can tease out not just the presenting complaint but can dive into the history and put the whole thing together in a complex way.

[Consultant 3, Int, 4‐year experience with PAs]

A PA participant noted that basic procedural skills were seen as beneficial for PAs' success postqualification. However, two PA participants felt restricted by a lack of hospital protocols despite having evidence of their skills.

basic procedural skills like taking bloods, blood gases and cannulation all contributed to my post qualification success…also gave me the appropriate clinical skills to be up and running quite quickly within the workplace.

[PA 1; Int, 6.5‐year experience]

A PA mentioned having the opportunity to develop additional procedural skills such as chest drains and arterial or central line insertions. A consultant suggested that the PAs should develop similar skills. Both a consultant and a PA interviewed emphasised the need to demonstrate ED skills through a portfolio.

I really like emergency medicine and would like to see a pathway recognising advanced skill practice perhaps with the Royal College of Emergency Medicine or Faculty of Physician Associates [FPA] portfolio, to show the additional skills and work toward them.

[PA 3; Int, 8.5‐year experience as a PA]

Doctors who participated in the online survey had varying opinions on the skills of PAs. Two doctors surveyed believe that PAs with experience develop diagnostic, investigative and management skills that are equivalent to many doctors.

From my limited experience with the physician associates, I feel that they are an asset to the multidisciplinary team with key diagnostic, investigative and management skills at the level of many of us doctors.

[FY2 4, OLS, 2‐ to 6‐month experience working with PAs]

The majority of the doctors on the online survey rated the PAs' ability to fulfil this task as excellent (42.9%) or good (39.3%; Table 4).

A consultant cautioned that it is dangerous to assume that PAs acquire clinical skills at the same level as junior doctors and emphasised that, although PAs are competent practitioners, they still require supervision.

… The danger is that it is assumed that all PAs attain the clinical skills of a junior doctor‐ some do but some don't, but all must be supervised however competent they are. PAs are an excellent addition to the ED workforce, but we must ensure that they ask for, and are given adequate supervision and support within a busy ED. What can sometimes can be forgotten is the additional workload for senior staff in training and supervising this group of PAs, whilst also supervising all the other junior clinical staff‐ a role that they have taken on additionally over the last few years on the shop floor when they oversee the clinical safety of all ED patients in the department.

[Consultant 7, OLS, > 1‐year experience working with PAs]

### 4.4 Scope of Practice of PAs

The versatility of PAs work within different department areas was mentioned across the data sets.

A PA mentioned that they work with flexibility within the ED and see undifferentiated patient cases.

… I work flexibly for example if the green zone is very busy or majors, I can go to work there and that might free up a senior to go to resus to see more sicker patients. Or if it is Resus that is very busy, I can go and help that doctor. I will see undifferentiated cases in the department … .

[PA 3; Int, 8.5‐year experience]

According to the online survey, 13 participants (46.4%) rated PAs' ability to handle undifferentiated patient cases as excellent, 12 (42.9%) as good, 1 as satisfactory and 2 as poor (Table 4).

Two experienced PAs and consultants interviewed mentioned a level of autonomy in two areas of the department.

I get quite a bit of autonomy. I can kind of run resuscitation.

[PA 1; Int, 6.5‐year experience as a PA]

The majority of participating doctors in the online survey rated the PA's ability to perform diagnostic and therapeutic procedures as excellent (42.9%) or good (25%) as shown in Table 4.

A consultant participant recognised that PAs have some level of autonomy, being left to manage certain areas of the ED with indirect oversight; however, the consultants also noted the need for PAs to be supervised.

… would generally say depending on who the person [PA] is, you know who they are and what extra supervision they would need, whether it is if you have to go to physically review things or not.

[Consultant 1; Int, 15‐year experience with PAs]

A PA mentioned that their dependency might be putting pressure on the doctors. A consultant interviewed, however, noted PAs not as being a burden, but rather saw them as supportive clinicians.

Although we [PAs] can do some things, we can't do certain things, so we might put pressure on the junior doctors, middle grade doctors or the consultants in taking their time to sometimes help with our patients … So that might be to do a senior review of a patient or it might be to offer a prescription.

[PA 4; Int, 10‐year experience]

However, one consultant expressed concern about the additional workload that PAs bring in terms of supervision. Four of the participating doctors and the PAs in the study were enthusiastic about the idea of regulating PAs so that they could eventually order investigations using ionising radiation and prescribe medication. This, they believed, would improve their work efficiency. Although a PA participant interviewed in one region noted an inability to prescribe medication as a frustration, it was not seen as a barrier as they have good working relationships with doctors to help with ordering and prescribing.

They [PAs] clerk the patient and examine the patient independently and come up with the differential diagnosis. Erm, then depending on the level of complexity, I will either just review the plans remotely and discuss the plan, or go and see the patient with them … . They should be authorised to request X‐Rays as this is needed to greatly enhance their performance and speed up patient's turnover.

[Consultant 2, Int, 5‐year experience working with PAs]

A consultant participant from another region thought that PAs' lack of such rights is a barrier as there is a refusal from some staff members to take responsibility for performing tasks on behalf of the PA.

… There are still people in my department who are still not taking them, or taking responsibility for ordering X‐Rays for them, mainly the nursing, like nurse practitioners who state that it is not in their job description to do that … so that is definitely a barrier.

[Consultant 1; Int, 15‐year experience with PAs]

A PA interviewed mentioned the inability to work at their full legal potential because of the lack of hospital protocols.

Unable to even work at my legal full potential due to trust level hindrances with protocols … Unable to order non‐ionising radiation even though other PAs in England can.

[PA 3; Int, 8.5‐year experience]

### 4.5 Professionalism of PAs

Two junior doctors participating in the online survey mentioned PA professionalism.

One doctor noted an example of a PA exhibiting less composure or discretion than a doctor would do.

… PAs are reminded of the need to respect and recognise Junior Doctors they work with. On at least one occasion, a very an otherwise competent PA wondered loudly why they should be seeing more patients but be said less than a middle‐grade doctor they were working with. This would not help the acceptance of this cadre … .

[SHO 5, OLS, 6‐month to 1‐year experience]

In addition to this, a doctor participant perceived PAs to be less professional on average.

… Slightly less professional on average in terms of keeping calm with colleagues, discretion. Somewhat looser norms in terms of working while tired or hungover.

[SHO 4, OLS 6‐month to 1‐year experience]

A patient participant mentioned a positive experience with the PA consultation included them appearing professional.

*[PA was]* professional, knowledagble and kept me informed.

[PA 27, PCQ]

## 5 Theme 2: Patient Recognition of PAs

### 5.1 Patient Perception of PA Role

**Figure 4:**
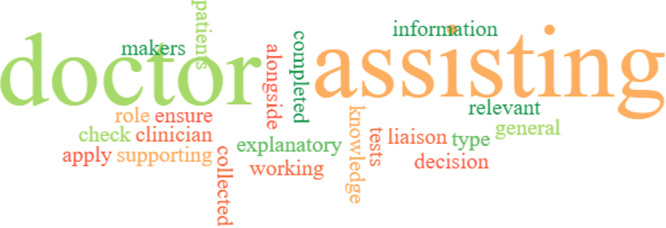
Word cloud (EDWordle.net) [29] generated from the patient responses regarding their understanding of the PA role. The size of the word is proportional to the number of times it was used to describe the PA role.

A word cloud was created using EdWordle.net (Beijing, China) [29] to display the participant patient's responses regarding their understanding of the PA role (Figure 4). The word cloud was created using the words used by the patient participants to describe the role of the PA. In the word cloud, larger words represented a higher frequency of use. The majority of the patients believed that the PAs are doctors or are there to assist the doctors.

A PA is a member of the team supporting doctors in examining patients, diagnosing and maintaining the care of patients. They carry out tasks that a doctor will complete**.**

[PCQ, Patient 23]

Assistant to physicians assisting in all areas and undertake work allocated by physicians to further the assistant's knowledge and experience.

[PCQ, Patient 27]

I'm not sure, thought she was a doctor.

[PCQ, Patient 13]

### 5.2 Impact of PA Personality and Characteristics

**Figure 5:**
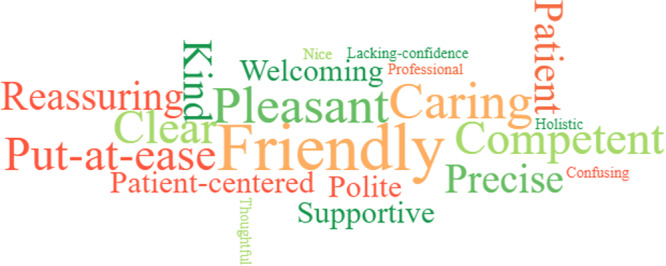
Word cloud (EDWordle.net) [28] generated from the varying words patient paticipants used to describe the PA they consulted with. The size of the word is proportional to the number of times it was used to describe the PA role.

A word cloud was created using EdWordle.net (Beijing, China) [29] to display the participant patient's perceptions of the PA in relation to their personality and characteristics (Figure 5). In the word cloud, larger words represented higher frequency of use.

*[The PA was]* very caring, answered all my questions, made me feel safe and reassured … .

[Patient 13, PCQ]

### 5.3 PAs Are Thorough and Detail Oriented

Fourteen patients surveyed noted that PAs were thorough in their consultations. A patient participant was surprised at the amount of time and attention the PA spent taking a full history, investigating and examining them, and ensuring that the correct diagnosis was made.

The PA role is to ensure that all relevant information has been collected and all tests have been completed during my visit. Bringing all the results together to make a plan for me going forward.

[Patient 7, PCQ]

From the online survey, 17 doctor participants (60.7%) rated the PAs' ability to provide health promotion and disease prevention advice as excellent, followed by 8 (28.6%) rating the PAs' ability as good (Table 4). Two doctors in this study noted PAs to be diligent; with one doctor also commenting on PAs' documentation to be thorough and complete.

… more diligent and have better documentation than most other doctors.

[Registrar 2, OLS, > 1‐year experience with PAs]

I was unaware of Physician Associates in the UK until I started working as an FY1—I have worked with PAs since including in ED as an F2. I have found PAs incredibly diligent and detail orientated members of the multidisciplinary team. I have learned a lot from the collective experience and have found them really great to work with … .

[FY2, OLS, 2‐ to 6‐month experience working with PAs]

### 5.4 PAs' Communication With Patients

Post consultation survey results with PA patients highlighted some positive experiences with the PAs' communication. Eight patient participants mentioned PAs to display active listening and five patient participants mentioned PAs to be good at keeping them up to date with their care. Eighteen patients mentioned positive experiences with their PA consultation that was related to the PA explaining their condition.

*[The PA]* explained everything very clearly and was very informative.

Four patient participants from the patient survey mentioned that the PAs were clear, and three said the PAs they saw provided reassurance.

Everything was clearly looked into regarding my visit for chest pain, and I was left with a clear idea of my prognosis.

[Patient 19, PCQ]

## 6 Theme 3: PAs Providing Continuity of Care

Participants across the data sets mentioned that PAs provide continuity of care to the department.

I am so happy with their presence, highly qualified, supportive, and more permenant where we establish a good rapport with them rather than junior doctors who rotate a lot.

[Registrar 2, OLS, > 1‐year experience working with PAs]

Established within the department, becoming a trusted force which stays within the department for years rather than moving every few months so become a repository for knowledge.

[Consultant 11, OLS, < 1‐year experience working with PAs]

A consultant interviewed noted that PAs help to maintain consistency in the department, as their familiar presence makes it easier to delegate tasks within the ED.

When turning up as a consultant and the team consists of 5 locums and a PA, you know the PA is a familiar face and consistent team members in the department so can deploy them accordingly.

[Consultant 2, Int, 5‐year experience working with PAs]

A PA interviewed expressed that being permanent staff members allows them to be familiar with the processes and help in orienting new staff members.

Continuity of my presence helps me to support the department helping to orientate doctors to help them settle into the ED; especially those who have only done ward work.

[PA 3; Int, 8.5‐year experience]

Two of the PA participants interviewed mentioned the benefits of being a continuous workforce in relation to relationships with the MDT and patient care.

I have built very good relationships with the MDT and with other clinicians in other specialities, in which sometimes means that I can get patient care facilitated quicker.

[PA 4, Int, 10‐year experience]

### 6.1 Confidence in and of PAs

A consultant interviewed appeared to have confidence in experienced PAs' abilities.

From our PAs there is a mixture of levels of where they are one of our experienced PAs which are absolutely fantastic, you wouldn't have any reason to doubt or double check anything, so very very good.

[Consultant 1; Int, 15‐year experience with PAs]

A doctor surveyed mentioned PAs confidence in their medical knowledge. A consultant interviewed noted more experienced PAs adhering to their scope of practice.

PAs are [an] integral part of the A&E, they work hard and has an important role in the ED. To a great extent they gain skills and experience easily, almost all of them have the confidence of medical knowledge and utilise it to provide above expectations care.

[Registrar 3, OLS, > 1‐year experience working with PAs]

I think in general they are good at saying that is not within my remit and I will need some support with this, especially the ones who have been there for a longtime, so that's less of a concern than maybe some of the doctors.

[Consultant 4, Int, 10‐year experience working with PAs]

Junior PAs were regarded by a consultant interviewed as being more hesitant and requiring more support. A patient participant reported a negative experience with the PA, noting that they appeared to lack confidence.

A little lacking of confidence which gave a slightly nervous manner.

[Patient 8, PCQ]

A doctor participant, however, noted PAs to be overconfident.

Overall fine, variable just like for doctors. Some are over confident, perhaps more commonly than with doctors of similar clinical experience.

[SHO 4, OLS, 6‐month to 1‐year experience working with PAs]

### 6.2 Providing Support to the Department

PA participants noted that they felt supported within their department and they equally supported their department for example assisting with the orientation of new staff or ensuring service delivery, while junior doctors attended teaching.

I help with ensuring service delivery when junior doctors in training go off the floor for teaching.

[PA 3, Int, 8.5‐year experience]

A consultant participant regarded PAs as playing a part in the smooth running of the ED.

They are extremely useful and help relieve the workload faced by clinicians, they are indispensable to the smooth running of the Emergency Department.

[Registrar 1, OLS, 6‐month to 1‐year experience working with PAs]

Three patient participants regarded the PA to be supportive

I assume the role is to support the work of busy physicians by doing some of the groundwork with the patients.

[Patient 10, PCQ]

### 6.3 Partnership and Teamwork Within the Department

One consultant noted that with the introduction of PAs to one region there were concerns about them replacing medical staff. However, it was soon understood that the PA role was complementary.

When they first came into the region there were some tensions between them *[PAs]* as it was thought that medical staffing would be replaced by PAs but people soon recognised that the roles are very different, more complementary and non‐antagonistic.

[Consultant 3, Int, 4‐year experience with PAs]

A PA also highlighted concern regarding the substitution of junior doctors with PAs in the ED on rotas because of an imbalance of skills.

I feel that the department loves us, but unfortunately they sometimes voted us in instead of junior doctors, and therefore their cannot always be an appropriate skill mix … We will have three or four PAs on the shop floor at one time, which is fantastic to have that workforce. But at the same time, we seem to have replaced a junior doctor on the rota.

[PA 4; Int, 10‐year experience]

There were multiple evidence across the data sets of PAs integration and work with colleagues in the ED. A PA interviewed perceived that they have good working relationships with the team through building trust with the MDT.

I have built up trust and good working relationships with doctors and nursing staff in the department. They trust my practice and judgment, this helps me to be efficient in my work.

[PA 4, Int, 10‐year experience]

A foundation doctor surveyed highlighted the positive experiences working with PAs.

I was unaware of Physician Associates in the UK until I started working as an FY1‐ I have worked with PAs since including ED as an F2. I have found PAs incredibly deligient and detail‐orientated members of the multidisciplinary team. I have learn a lot from the collective experience and have found them really great to work with. I certainly support their professional development and presence and would welcome to see more across the workforce. Many thanks for your collective contribution to excellent patient care.

[SHO 2, OLS, > 1‐year experience working with PAs]

A consultant stated that PAs work alongside them to see patients more efficiently, and a patient participant also perceived PAs to work alongside the doctors.

… you know what their capabilities are, so you can deploy them, accordingly, whether that means being your pair of hands and you both work together to see patients quickly or working alongside them and lead them with the paperwork and implementing them.

[Consultant 2, Int, 5‐year experience working with PAs]

Working alongside the doctors, being more explanatory and helpful with more options.

[Patient 5, PCQ]

A level of trust in the PAs' competence was implied when a consultant participant noted that PAs were conducting the ward round in a section of the department and alerting the consultants of the patients who require prioritisation.

PAs can run the clinical decisions unit (CDU) or observation ward, doing ward rounds and support the red flag checking of patients.

[Consultant 4, Int, 2‐year experience working with PAs]

## 7 Theme 4: Future PA and Registration

### 7.1 Future Steps for the PA Role in the ED Setting

A consultant interviewed discussed the training and work dynamics relating to PAs progression, burnout and job satisfaction.

… a GP trainee would train for months in the the ED and come with an agenda of how does the ED work for me as a GP in the future. Where as the PAs in the department work with the focus on ED, but the difficulty is how do you get that progression and prevent the burnout? As it is relentless at the moment and I think that is the real challenge at the moment … 37.5 hours a week of just taking the next patient out of the majors’ pile, clerking, doing the paperwork and referring on wouldn't give me job satisfaction.

[Consultant 2, Int, 5‐year experience working with PAs]

A senior PA participant interviewed mentioned that there is a fear of attrition because of burnout. Despite this, two consultants across the data sets reported their departments to have high PA retention rates.

I feel like we have a high turnover of PAs, not as high as it used to be, but I feel like after 3‐4 years in the ED, there can be a bit of burnout.

[PA 4; Int, 10‐year experience]

Three PAs interviewed mentioned that the lack of progression and development were negative aspects of their role.

Lack of progression and development … No progression, stuck at the top of band 7.

[PA 1; Int, 6.5‐year experience]

All four consultants interviewed, two doctors online and all four PAs interviewed also mentioned the need for prescribing rights and ordering ionising radiation.

### 7.2 Role Combination

A participant PA interviewed mentioned a way in which the PA profession could develop is via combining the role with pre‐existing professions, that is, paramedic science working to prevent patient admissions. Another PA interviewed suggested the creation of fellowships such as leadership or teaching fellowships. They also suggested making use of secondments into different areas of the hospital such as the Intensive Therapy Unit (ITU) for skill advancement, similar to the experience another PA participant from a different region had the opportunity to fulfil.

Development roles; so for example, I was thinking we could have a PA who is almost like a teaching fellow; and could have a PA who is a leadership fellow … more ability to learn and practice clinical skills. At the moment we have a PA who has joined us from [USA] who is obviously got really big skill mix with regards to doing central lines, lumbar punctures. Us UK ED PAs never really had the opportunity to do those skills in the ED; so either clinic time that is just focused on clinical skills or rotations into places like ITU or ambulatory care where PAs can constantly practice more clinically skills is another idea for development.

[PA 4; Int, 10‐year experience]

A consultant interviewed suggested more transparency with PA procedural skills attainment across regions. Four doctors across the data sets suggested that the PA role could be developed alongside training to become a doctor as opposed to going back to study graduate entry medicine for those who may want to be in a more independent role.

I've met a few PAs who said they have completed PA studies in a hope to do graduate entry medicine … .I am not sure that this is a good use of time. I think it would be interesting as registration comes in I suspect there would be some level of advancement or training and converting into medicine on the job.

[Consultant 2, Int, 5‐year' experience with PAs]

### 7.3 Future Workforce Potential

During the interviews, a consultant participant expressed difficulty in convincing others to recruit PAs into the ED. They believed that some clinicians preferred experienced professionals who were ready to start working immediately. The lack of prescribing rights and inability to order ionisation radiation, in addition to being less established than ACPs, were seen as hindrances to recruiting PAs.

It is difficult to employ more PAs in our ED. We have two posts going for PAs or ACPs, but I am wary about how we are going with this next recruitment as I know upstairs is pushing for ACPs, but I want a PA also, not just ACPs. I don't want two the same, but the difficulty is locally you have more people ready to do the ACP role than there are PAs. And frequently what you get with PAs is having to start from the beginning again. Although I personally feel it is worth the investment, not all of my colleagues do, they instead want somebody who is ready to run. That's why I strongly feel a foundation programme for PAs will make them ready to run so they will come on a level footing in fact probably a better footing than ACPs.

[Consultant 1, Int, 15‐year experience working with PAs]

There was a suggestion of creating a postqualification programme in emergency medicine by two consultant participants to address this issue by a consultant interviewee.

A lack of clear progression of a PA is also of concern to some consultants. There was a suggestion of PAs completing a foundation programme or preceptorship in Emergency medicine post qualifying to help with their employability in the ED.

[Consultant 2, Int, 5‐year experience with PAs]

Furthermore, financial constraints were mentioned. PAs were considered expensive to recruit in one region by a consultant participant, whereas a junior doctor participant suggested that PAs were a cheaper alternative to doctors.

The other thing is that they are quite expensive. Like for like they are a great asset, but they are not a cheap replacement for doctors and I don't think they were ever meant to be. Some people try to view them like that and I don't think economically that stacks up.

[Consultant 3, Int, 4‐year experience working with PAs]

### 7.4 Governance

In this study, participant consultants from two regions noted that PAs are paid under the Agenda for Change (AfC) pay system designed for nondoctor and nondentist NHS staff. They note PAs are considered part of the nursing hierarchy for certain purposes, and the medical team manages their clinical work. The same two consultants interviewed emphasised the need for a unified governing structure for PAs and moving away from AfC in the future.

Over here they do not fall under medicine, instead they are under the nursing agenda for change. Their responsible director isn't medicine, they fall under the nursing governance structure. So while they report to me clinically, if there are other issue I have to go through a different division and structure. Just simple stuff trying to sort out paperwork for them or I know its never happened but if there is a disciplinary issue it will be a completely different process. I hasten to say this has never happened, but I am aware of it.

[Consultant 3, 4‐year experience working with PAs]

I think as well from a medical manager managing PAs, difficulties is that they are on Agenda for change, they are not on a medical management structure. So all their kind of like management stuff doesn't come from me. So are PAs although clinically we manage them, if they are absent, any disciplinary comes through the nursing hierarchy agenda for change. I think that's a major disadvantage and I don't think there's a way of fixing that but I actually think they should be in the medical management structure.

[Consultant 1, Int, 15‐year experience working with PAs]

Additionally, both consultants and two doctors from the online survey mentioned the regulation of PAs by the GMC.

I think PAs are a valuable asset to our ED. They are able to work within the team to manage patients appropriately. I believe that once formal registration with the GMC is in place that this will enhance their role in the ED.

[Consultant 9; OLS, > 1‐year experience working with PAs]

## 8 Discussion

The objective of this study was to examine how ED doctors, patients and PAs perceive the role of PAs in the UK EDs. The study identified four themes (PAs are fit for purpose; patient recognition of PAs; PAs providing continuity of care; and the future PA and regulation) that were classified under the four domains of the GMC Good Medical Practice prerequisites: professional values and behaviours, including knowledge, skills and development; patients, partnership and communication; colleagues, culture and safety; and trust and professionalism. Additionally, the study identified other subthemes such as training, clinical knowledge, clinical skills, scope of practice, professionalism, communicating with patients, personality and characteristics, thoroughness, confidence in and of PAs, providing support to the department, interaction with other staff members, future steps for the PA role in the ED, role combination, future workforce potential and governance. These findings provide insight into the perceptions of different groups regarding the PA role in the UK EDs.

**Figure 6:**
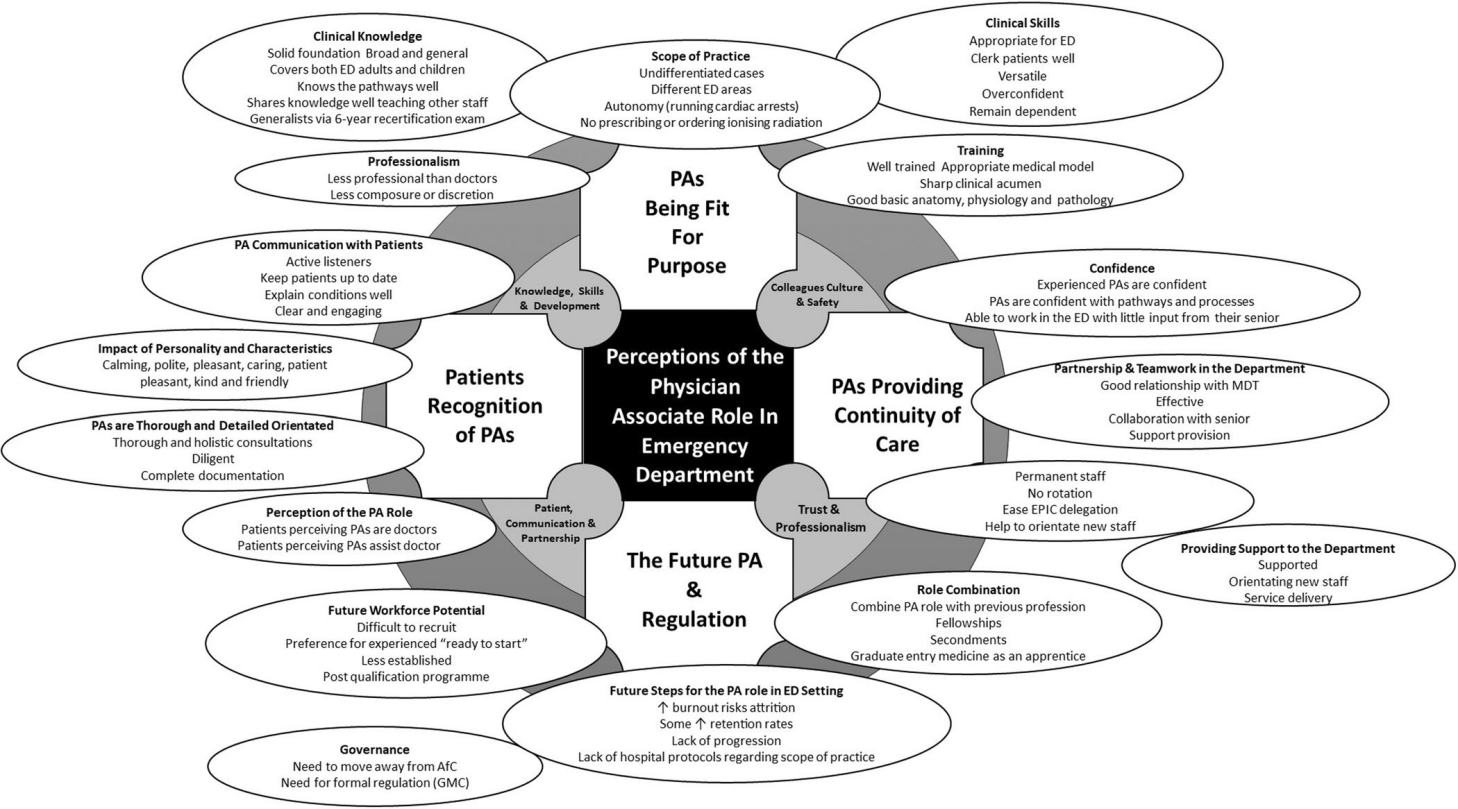
Representation of the interlinking perceptions of the physician associate role in the UK ED. AfC, Agenda for Change; ED, Emergency Department; GMC, General Medical Council; PA, physician associate. Clerk **=** to perform a thorough medical examination, record the findings in the medical notes, and develop a comprehensive diagnosis, problem list and care plan.

Figure 6 displays a summary of the findings from this study according to the themes and subthemes.

### 8.1 Domain 1: Knowledge, Skills and Development

The PA medical model training was mentioned in this study as being appropriate for the ED, as opposed to the nursing care model used interchangeably for advanced nurse practitioners or ACPs. This comparison has been made in previous studies [30]. PAs' clinical skills were regarded as being versatile within the department, which has also been mentioned in previous studies [31, 32, 33]. From this study, there appears to be a need for transparency across the regions in terms of hospital protocol to enable all PAs to work at their current scope of practice. The GMC governance sets to develop a full, clear and robust national guidance for PAs' scope of practice [34]. This will hopefully help to address this discrepancy by ensuring the PA's role and responsibilities are clearer to all.

Although PAs' training is said to be appropriate for the ED, there has been a discrepancy between a newly qualified and experienced ED PA as noted previously [35]. Two of the regions that had less experience of PAs and a lower number of PAs working in their department highlighted the need of getting PAs up to speed to improve employability within the department. It was suggested that this could be achieved via the completion of a postgraduate foundation ED programme. Such a programme exists within the Royal College of Emergency Medicine (RCEM) currently running in six sites across India and Pakistan for doctors [36]. Perhaps this programme could be extended to include PAs and ensure an introduction to additional clinical skills and protocols are in place and evidenced during that time. Some patients and doctors in our study mentioned how thorough PAs were as clinicians. There was a comment about their clear documentation. Other studies have also found PAs' documentation to be thorough [21, 37, 38] and there was a suggestion that the ED metrics may be affected because of the level of PA documentation [39]. Our study could have been enhanced if combined with impact metrics [40] and or included observations of case notes [39] to better experience the level of PA patient documentation and its effects on the department throughput.

### 8.2 Domain 2: Patients, Partnership and Communication

Interestingly, patients expressed high levels of satisfaction with consultations with PAs, with 96.5% of patients willing to see a PA in the future. This is consistent with other studies internationally, such as Jeanmonod et al., where 91.6% of the 6639 ED patients surveyed in an American ED were willing to be seen by a PA in the future [37], and nationally Taylor, Halter and Drennan's study, which surveyed 273 ED patients and revealed that 88% of them were satisfied with the PA encounter [42]. Effective communication has been linked to high levels of patient satisfaction with health professionals, who could account for the reason why patients surveyed in this study were satisfied with the care received from PAs [6]. In the future, it would be useful to link the outcomes of patients in the ED to the level of satisfaction. According to our study, some patients mistakenly believed that they were being treated by a doctor. The misconception of PAs as doctors has also been noted in other UK studies [38, 39, 40, 41]. Despite the PA role being present in the United States for over 60 years, a survey conducted in 2021 showed that the PA role confusion is longstanding with 25% of patients wrongly believing PAs to be doctors [43]. Patients surveyed appear to have a level of trust in PAs, but a lack of comprehension regarding their duties can result in reduced trust and satisfaction because of communication breakdown [44]. To combat this, it is crucial to continuously enhance communication about the PA's role and confirm patient comprehension during consultations. The FPA has issued guidance on this matter [45]. In this study, the PAs wore the same colour scrubs as radiographers and doctors, which may have contributed to patient confusion. Previous studies have demonstrated that nonverbal signals, such as uniform scrub colours, can influence communication and perception among patients and staff [46]. Further studies looking at the process of communication such as de Haes and Bensing [47] and Taylor, Halter and Drennan's study [42] would be beneficial to identify specific areas of possible communication breakdown between the PA and patient.

### 8.3 Domain 3: Colleagues, Culture and Safety

The PA role is said to add longevity and continuity to the ED due to being permanent staff members; the benefits highlighted in this study of the Emergency Physician In Charge (EPIC) being aware of their skill mix at the beginning of the shift, having confidence in the PAs' abilities and capabilities and the PAs themselves helping with orientation of junior staff members early on within their rotation. Such discoveries have been noted in previous studies [48, 49, 50, 51, 52]. However, there is a noted worry of burnout because of the physical and mental demands that come with the ED job. This ultimately risks attrition as mentioned by consultants and a lead PA within this study. Burnout of ED PAs has been studied in a previous study and was linked to poor supervision [53]. There are many support networks for health professionals currently available, such as ‘Doctors In Distress’ that provides all health professionals with a space to speak confidentiality about the emotional impact of their job in the hope to reduce the number of work stress‐related suicides among medical professionals [54].

PAs remain generalists within a specialist area, and it was mentioned in this study that the recertification exam took six yearly aids in maintaining this. However, this exam has been abolished since 2022 and will be replaced by a new e‐portfolio with the GMC [55]. There will however still be opportunities to continue to demonstrate generalism via various means such as reflective accounts from Continual Professional Development (CPD) courses, secondments or role combinations and experiences [55].

### 8.4 Domain 4: Trust and Professionalism

In this study, experienced PAs were observed to be trusted to work in higher acuity areas and to recognise which patients require prioritisation. Another study has noted PAs to consult more with specialities and refer less when compared with ED Physicians [48]. According to our study, the permanent roles of the PAs have established trust between their seniors. This trust has been built over time because of the PAs' consistent and reliable presence in the workforce, which has demonstrated their abilities and capabilities. In our study, it was emphasised that despite PAs providing a stable workforce, it is important to recognise them as a separate workforce from doctors and not as substitutes.

#### 8.4.1 Study Strengths, Limitations and Future Studies

Our study was strengthened by incorporating views from four regions of the United Kingdom. However, our study was limited because only one PA and one consultant from each region were interviewed. However, the recruited clinicians were considered the most senior in their respective groups, which provided a better chance of obtaining more experienced viewpoints regarding the NHS systems. The views expressed by the participants in the present study were based on their personal experiences and so were likely to be influenced by the characteristics of the individual PAs they interacted with at the time. Therefore, responses may differ from others' experiences across different ED sites. It is important to note that this study was relatively small in scale, and therefore, the views expressed in it should not be considered as representative; instead, they should be seen as a starting point for further research. The study relied on data collected from a single online survey and post consultation questionnaire which was conducted on a UK site hosting the largest number of ED PAs over a long period. However, it is worth noting that because the main hospital site has a well‐established PA team, the views expressed in the study may differ from those of more newly established PA teams. More careful wording and field testing of the online survey were required in an attempt to minimise any response bias. Although patients were purposefully selected as being seen by a PA, they were self‐selecting in the sense that there was no control over their demographics or conditions they were being seen. However, they did represent diversity within their responses. It was clear that the interviews produced more favourable opinions regarding PA in contrast to the online survey, which presented more varied responses. It is possible that participants felt hesitant to express negative views during the interview because of the interviewer's insider status. To enhance the robustness of this study, it would have benefited from incorporating quantitative impact PA ED metrics data. Given the availability of funding, research study nurses could have been deployed to facilitate the recruitment and collection of post consultation patient questionnaire data. This would have helped to separate the insider researcher from those being researched.

This study was conducted 2 years ago. If the study were to be updated, it is likely that there would be more mixed views and opinions about the use of PAs because of the rising concerns about patient safety [56]. In any future replication of this study, increased participants should be considered for the online survey, patient satisfaction questionnaire, and virtual interviews to augment the breadth and depth of the information gathered.

## 9 Conclusion

Perceptions of a new healthcare professional role are important for understanding their overall acceptability and for appropriate integration of the role within the health service. With all clinicians, there were varying perceptions based on the PAs' levels of experience; however, they should be regarded as a distinct workforce. There were many positive viewpoints on the ED PA role reported by ED doctors, patients and PAs between February and May 2022; there were also some dissenting opinions, and some areas of concern were raised, such as overconfidence and the level of supervision required. The PAs' ‘being fit for purpose’ was a common theme. With time, the benefits of the continuity of care the PAs provide the department can be observed as noted by this study. However, the perceptions collated within this study raised an issue around the need for increased awareness of the PA role among patients; perhaps through improved patient–clinician communication and nonverbal clues such as uniform recognition. Increased awareness of the PA scope of practice within the ED was another area needing more development, especially to ensure the PA scope aligns with the hospital's protocols, better enabling the PA to work to their full potential in the ED within set boundaries. From the perceptions within this study, there was also a clear need for a more robust career progression pathway for PAs with a more aligned management and remuneration structure. With the suggested uptake of an ED programme for newly qualified PAs entering the ED and statutory regulation, PA employability within the ED would increase. The findings of this study offer valuable insights into the perceptions of patients, doctors and PAs regarding the role of PAs in emergency medicine. These insights could prove beneficial to various stakeholders such as the GMC, which will soon be regulating the profession, as well as human resources and education faculties.

## Author Contributions

**Nicole M.A. King:** conceptualisation, investigation, methodology, formal analysis, writing–original draft, writing–review and editing, visualisation. **Suzannah Helps:** supervision, writing–review and editing. **Yee Gan Ong:** writing–review and editing. **Sandra Walker:** supervision, formal analysis, writing–review and editing.

## Ethics Statement

Ethical and research governance approval for this study was obtained from the Research Ethics Committee (REC Reference: 221/PR/0802). Informed consent was obtained from the patients, doctors and PAs who took part in the study.

## Consent

Informed consent was obtained from the patients, doctors and PAs who took part in the study.

## Conflicts of Interest

Nicole M. A. King is currently employed as an Emergency Medicine physician associate.

## Supporting information

Supporting information.

Supporting information.

Supporting information.

## Data Availability

The data that support the findings of this study are available in the Supporting Information Material of this article.
