# Supplementary material for: Doctors', Patients' and Physician Associates' Perceptions of the Physician Associate Role in the Emergency Department
Source: Health Expect. 2024 Jul 10;27(4):e14135. doi: 10.1111/hex.14135 (PMC11233990; doi:10.1111/hex.14135)
Supplement: Supplementary file 1 — Supporting information. [file HEX-27-e14135-s001.pdf]

Epsom and St Helier University Hospitals NHS Trust

UNIVERSITY OF PORTSMOUTH

Dear patient, thank you for taking part in the IMpact And Perceptions of the Physician Associate Role in the Emergency Department (MAPPED Study)

We would be grateful if you would complete this questionnaire about your visit

Please answer all the questions below. There are no right or wrong answers and your opinion is highly valued

Kindly hand the completed questionnaire in the envelope provided to the Emergency Department reception team

1.You were seen by a Physician Associate. What do you understand their role to be?

____

____

____

____

2. How would you rate the care you received from the Physician Associate? (Please tick your response)

Poor

Fair

Good

Excellent

3. What was positive about your experience with the Physician Associate?

____

____

4. What was negative about your experience with the Physician Associate?

____

____

5. Would you be keen to be seen by a Physician Associate in any future attendances to the Emergency Department? (Please tick your response)

Yes

No
