# Supplementary material for: Doctors', Patients' and Physician Associates' Perceptions of the Physician Associate Role in the Emergency Department
Source: Health Expect. 2024 Jul 10;27(4):e14135. doi: 10.1111/hex.14135 (PMC11233990; doi:10.1111/hex.14135)
Supplement: Supplementary file 2 — Supporting information. [file HEX-27-e14135-s003.docx]

**COREQ (COnsolidated criteria for REporting Qualitative research) Checklist**

A checklist of items that should be included in reports of qualitative research. You must report the page number in your manuscript where you consider each of the items listed in this checklist. If you have not included this information, either revise your manuscript accordingly before submitting or note N/A.

| **Topic** | **Item No.** | **Guide Questions/Description** | **Reported on Page No.** |
| --- | --- | --- | --- |
| **Domain 1: Research team and reflexivity** |  |  |  |
| *Personal characteristics* |  |  |  |
| Interviewer/facilitator | 1 | Which author/s conducted the interview or focus group? **Main** **Researcher** | 8, 10 |
| Credentials | 2 | What were the researcher’s credentials? E.g. PhD, MD **PhD** | 1 |
| Occupation | 3 | What was their occupation at the time of the study? **Emergency Medicine Physician Associate** | 50 |
| Gender | 4 | Was the researcher male or female? **Female** |  |
| Experience and training | 5 | What experience or training did the researcher have? **Good Clinical Practice (GCP) trained. The NHS Ethics approved** | 6 |
| *Relationship with participants* |  |  |  |
| Relationship established | 6 | Was a relationship established prior to study commencement? **The only prior interview contact was via the main researcher’s communicating with the participants for recruitment, consenting and scheduling of the interviews** | 6 |
| Participant knowledge of the interviewer | 7 | What did the participants know about the researcher? e.g. personal goals, reasons for doing the research. **Information about the study was contained within the email advertisement and on the information sheet, sent to the participants via email. The aim was also repeated on the virtual interview ahead of the questions as was a statement about the interview being recorded, transcribed verbatim within 10 days, then deleted.** | 6 |
| Interviewer characteristics | 8 | What characteristics were reported about the interviewer/facilitator? e.g. Bias, assumptions, reasons, and interests in the research topics  **PAs being a relatively novel profession, little literature surrounding their role and perceptions in the ED; the main researcher’s insider status and need for a reflexive lens on throughout.** | 6,8,13 |
| **Domain 2: Study design** |  |  |  |
| *Theoretical framework* |  |  |  |
| Methodological orientation and Theory | 9 | What methodological orientation was stated to underpin the study? e.g.  grounded theory, discourse analysis, ethnography, phenomenology, content analysis  **A variation of Braun & Clarke’s Thematic analysis- hybrid thematic analysis informed by Schutz’s theory of social phenomenology. The inductive approach was underpinned by Boyatiz and the a priori themes approach by Crambtree & Miller.** | 10-11 |
| *Participant selection* |  |  |  |
| Sampling | 10 | How were participants selected? e.g. purposive, convenience, consecutive, snowball. **Purposive sampling** | 8,17 |
| Method of approach | 11 | How were participants approached? e.g. face-to-face, telephone, mail, email. **Via secure webmail** | 8 |
| Sample size | 12 | How many participants were in the study? **4 ED consultants and 4 ED PAs** | 1,17 |
| Non-participation | 13 | How many people refused to participate or dropped out? Reasons? **100% of the set purposive sample took part. No dropouts.** | 17 |
| *Setting* |  |  |  |
| Setting of data collection | 14 | Where was the data collected? e.g. home, clinic, workplace **Home/Workplace** |  |
| Presence of nonparticipants | 15 | Was anyone else present besides the participants and researchers? **Only the main researcher and the participant at any one time** |  |
| Description of sample | 16 | What are the important characteristics of the sample? e.g. demographic data, date  **Three male, 1 female ED Consultant; 3 male PAs and 1 female ED PA.**  **ED Consultants worked with PAs for an average of 8.5 years; ED PAs has been qualified for an average of 7.1 years. 3/4 PAs worked in the ED since qualifying; ¼ from a T&O/ED internship.** | 17 |
| *Data collection* |  |  |  |
| Interview guide | 17 | Were questions, prompts, guides provided by the authors? Was it pilot tested? **Semi-structured interviews –set questions based around the standard GMC colleague satisfaction survey** | 9 |
| Repeat interviews | 18 | Were repeat inter views carried out? If yes, how many? **No** |  |
| Audio/visual recording | 19 | Did the research use audio or visual recording to collect the data? **Visual recorded/Virtual interviews via Microsoft Teams** | 10 |
| Field notes | 20 | Were field notes made during and/or after the inter view or focus group? **The recording was listened to and transcribed verbatim within 10 days of the interview.** | 10 |
| Duration | 21 | What was the duration of the interviews or focus group? **10-15 minutes** | 8 |
| Data saturation | 22 | Was data saturation discussed? **100% of set purposive sample** | 17 |
| Transcripts returned | 23 | Were transcripts returned to participants for comment and/or correction **No** | 13 |
| **Topic** | **Item No.** | **Guide Questions/Description** | **Reported on Page No.** |
|  |  |  |  |
| **Domain 3: analysis and findings** |  |  |  |
| *Data analysis* |  |  |  |
| Number of data coders | 24 | How many data coders coded the data? **Two** | 10-12 |
| Description of the coding tree | 25 | Did authors provide a description of the coding tree? **Yes** | 12,18 |
| Derivation of themes | 26 | Were themes identified in advance or derived from the data? **Both via hybrid thematic analysis** | 1-2, 10-12 |
| Software | 27 | What software, if applicable, was used to manage the data? **Microsoft Excel** | 11 |
| Participant checking | 28 | Did participants provide feedback on the findings? **No** | 13 |
| *Reporting* |  |  |  |
| Quotations presented | 29 | Were participant quotations presented to illustrate the themes/findings?  Was each quotation identified? e.g. participant number **Yes; (Doctor, PA, number, data set, number of years worked with PAs)** | 16,19-37 |
| Data and findings consistent | 30 | Was there consistency between the data presented and the findings? **Yes** | 38-42 |
| Clarity of major themes | 31 | Were major themes clearly presented in the findings? **Yes (PAs being fit for purpose, Patient recognition of PAs, PAs providing continuity of care and The future PA and regulation)** | 17, 19-37 |
| Clarity of minor themes | 32 | Is there a description of diverse cases or discussion of minor themes? **Subthemes are displayed in Figure 3 and described in the results sections under the respective main themes** | 18--37 |

Developed from: Tong A, Sainsbury P, Craig J. Consolidated criteria for reporting qualitative research (COREQ): a 32-item checklist for interviews and focus groups. *International Journal for Quality in Health Care*. 2007. Volume 19, Number 6: pp. 349 – 357

**Once you have completed this checklist, please save a copy and upload it as part of your submission. DO NOT** **include this checklist as part of the main manuscript document. It must be uploaded as a separate file.**
