# Supplementary material for: Doctors', Patients' and Physician Associates' Perceptions of the Physician Associate Role in the Emergency Department
Source: Health Expect. 2024 Jul 10;27(4):e14135. doi: 10.1111/hex.14135 (PMC11233990; doi:10.1111/hex.14135)
Supplement: Supplementary file 3 — Supporting information. [file HEX-27-e14135-s002.docx]

**Supplementary Information 3: Themes and additional illustrative quotes from the Patient Post Consultation Questionnaire (PCQ), Doctors and Physician Associate virtual Interviews (Int) and the Doctor’s online survey (OLS).**

| **Theme 1: PAs being Fit for Purpose** | |
| --- | --- |
| **Sub theme** | **Illustrative quotes** |
| Training of PAs | *“Enables a bit more of lateral thinking approach rather than that fixed mind set of my paramedical science training”* [PA 2; int, 3 years’ experience]  *“we* [PAs] *got a good basis in terms of managing and investigating diseases. Also, we go our intermediate Life Suport Qualification whilst doing the course as well. So that contributed to getting my job”* [PA 1; int, 6.5 years’ experience]  *“I wouldn’t say PAs are much better I would say they are equivalent with the same training and they shouldn’t be discriminated against for being a PA rather than being a doctor or ACP. But a lot of people and scared of the physician associate it’s got to be a doctor or it’s got to be an ACP because they are more established in the UK because they come from a nursing background”* [Consultant 1; Int, 15 years’ experience with PAs]  *“There's always a bit of a fight going on between use of the PA against ACP and I will be honest, I feel that the PA role is preferable. Because of the PAs training model, yes, it is a shorter course, but it is a kind of medical model of training. So you actually could it be trained to actually fill up the role of our frontline clinician from the beginning. PAs training is appropriate and the right training for the ED”* [Consultant 1; Int, 15 years’ experience with PAs]  *“I believe they are not only adequate but also very well qualified and efficient. They are no less than any other doctor in the department. Their presence is absolutely crucial for the safety of patients as well as the smooth running of the department”* [SHO 1; OLS >1 year working with PAs].  *“ despite the necessary training in pharmacology, therapeutics with evidence in my clinical skills log book as a record of competency; I am unable to use it due to a lack of trust protocol”* [PA 3; int, 8.5 years’ experience]  *“The other barrier I think there is a bit of a gap, is how do we get someone who is finished PA school who has done their training there to develop into a clinician via an apprenticeship, because it is a massive jump. It is almost like a medical student going to be an SHO without doing their foundation years. I think there needs to be a foundation programme for PAs”* [Consultant 1; Int, 15 years’ experience with PAs] |
| Clinical Knowledge of PAs | *“* [PAs] *apply a general knowledge in an A&E setting*”[ Patient 8, PCQ].  *“..She knew to contact regarding my issue”* [Patient 32, PCQ]  *“She understood my condition”* [Patient 43, PCQ]  “*Excellent explanations about what was happening to me*” [Patient 52, PCQ]  “[PA] *understood what I said and advised for an X-Ray”* [Patient 3, PCQ]  “*Good knowledge of my problem, well explained”* [Patient 6, PCQ].  *“ Speaking to the PAs the revalidation exam taken every 6 years helps keep their knowledge up to date and the ED is a great general specialty to enable that”* [Consultant 2, Int, 5 years’ experience with PAs]  “ *The knowledge, experience and kindness shown was a brilliant experience, thank you*.” [Patient 39, PCQ].  “ [PA] *very friendly, polite and reassuring”* [Patient 15, PCQ].  “ She [PA] *was helpful, kind and reassuring”* [Patient 21, PCQ].  *“A more experienced PA I would put on par with not maybe a registrar, but perhaps a step below that with their knowledge in the department”* [Consultant 1; Int, 13 years’ experience with PAs]  *“…via the GMC that is how I think they will develop as they will become more independent and autonomous practitioners equivalent to an emergency nurse practitioner and advanced nurse practitioner but with additional medical stances*.*”* [Consultant 1; Int, 15 years’ experience with PAs]  *“..they brought an awful lot of knowledge and expertise to the department. It wasn’t something I knew particularly much about . I knew the role existed but not what they actually did. I got a rapid steep learning curve and I am delighted that I have Physician Associates in the department”* [Consultant 4, Int, 4 years’ experience working with PAs]  *“The majority is hardworking with excellent knowledge of emergency medicine”* [Middle grade 2, OLS, >1 year experience working with PAs]  *“…more solid foundation of knowledge, willingness, and ability to keep knowledge and practical skills up to date”* [Registrar 2, OLS, >1 year experience with PAs]  *“I* [PA] *support the department by providing teaching to ANPs, PAs and students including medical students”* [PA 1; int, 6.5 years’ experience] |
| Clinical Skills of PAs | *“PAs have the appropriate clinical skills and training to come and deliver patient care in the ED”* [Consultant 1; Int, 15 years’ experience with PAs]  *“PAs are good at investigating patient’s backgrounds and previous histories”.* [Consultant 2, Int, 5 years experience working with PAs]  *“Again they are equivalent. To me they work at F2/ST1 level like any profession there is an array of capabilities but they seemed to be trained to a very good standard and their ability to take a history, perform an examination and come up with management plans is excellent****”***[ Consultant 3, Int, 4 years experience working with PAs]  *“They are playing the most important part to deliver the best possible service for the unwell patients who present to the ED”*[ Consultant 10, OLS, >1 years’ experience working with PAs] |
| Scope of Practice of PAs | “*I like the vast array of different orientations we see a range from all sorts of minor injuries to major patients to whatever patient we get coming through. We are quite a busy emergency department, so I like the breath and wide variety of patients we see really*” [PA 2; int, 3 years’ experience]  *“ the inability to prescribe medication is a frustration, but not a barrier. I have built up trust to ask a doctor for the prescription post giving reasoning. The main barrier is administration of medication*” [PA 3; int, 8.5 years’ experience]  “*We don’t have free rein obviously we practice under the scope of our title*’. [PA 2; int, 3 years’ experience]  “*I think they need prescribing rights and EMER/radiology rights but I know there is work being done about this via the GMC*” [Consultant 3, Int, 4 years' experience working with PAs]  “ *I have to get the medication prescribed by a doctor, but I am unable to administer it, so have to go and get a nurse to give the medication, but they might be very busy; delays patient’s treatment* ”[PA 3; int, 8.5 years’ experience]  “*Lack of prescribing rights and ordering ionising radiation and lack of a regulating body is a barrier to their full performance* [Consultant 1, Int, 13 years' experience working with PAs] |

| **Theme 2: Patient Recognition of PAs** | |
| --- | --- |
| **Subtheme** | **Illustrative quote** |
| PA Communication with Patients | *“.. she explained everything and spoke to me as a patient and not just to my daughter...”* [Patient 24, PCQ]  *“ I was kept up to date with what is going on with my symptoms and treatment*” [Patient 50, PCQ]  *“Explained everything so perfectly”* [Patient 46, PCQ]  “*Clear explanation of my diagnosis. I was taken seriously when describing my symptoms. X was very friendly and relatable*” [Patient 41, PCQ]  “*I understood everything* x *said as* x *explained all what he was doing and why*..” [Patient 42, PCQ]  “ *X was very attentive and listened to my concerns. Helped to get to support needed*. [Patient 45, PCQ]  “*X listened and asked open questions*”[Patient 57, PCQ] |
| PAs are Thorough and Detailed Orientated | *“ This role was created to ensure that the medical structure is even more tight in ensuring patient care is thorough and professionally managed”* [Patient 38, PCQ]  *“the things you can’t discuss with a doctor maybe because of lack of time, can be done with an associate physician”* (Patient 5, PCQ)  *“ I was asked about my symptoms plus pre-existing condition, PA explained what tests/X-ray he would request after a very thorough examination”* [Patient 23, PCQ]  *“ she took time to really appreciate some of the non-presenting issues such as my fluid intake and recognised that I needed some water and got me some”* [Patient 38, PCQ]  *“PAs take thorough histories from the patients and think outside of the box”* [Consultant 4, Int, 4 years’ experience working with PAs] |
| Patient Perception of the PA Role | “*To take medical histories, perform examinations and analyse test results* ” [Patient 34, PCQ]  “ *I didn’t understand what an associate physician meant, I thought x was doctor*” [Patient 41, PCQ]  “[PAs are] *there to assist doctors”*[Patient 1, PCQ]  “*Assisting doctors and decision-makers*” [Patient 6, PCQ]  “*similar to a junior doctor*” [Patient 15, PCQ]  “*To obtain details as to why I found it necessary to visit A&E. To arrange for me to receive the necessary and appropriate treatment*”[Patient 14, PCQ]  “*As doctors are very busy, physician assistants are trained to take the load off the doctors*”[Patient 53, PCQ]  “*I’m not sure what the difference is between various roles. However, from our conversation it is clear that there is a monitoring programme in place for junior/newly appointed staff. This clearly provides a learning experience without the risk*” [Patient 16,PCQ]  “*A person considered to be between a qualified nurse and a doctor*.” [Patient 19, PCQ]  “ *To assess and consult with doctors*”[ Patient 33, PCQ] |
| Impact of PA Personality and Characteristics | “Very *competent and thoughtful*” [Patient 9, PCQ]  “*Very friendly, polite and reassuring*” [Patient 15, PCQ]  “*X was very nice, caring and patient with me. Did make me feel at ease*”[Patient 20, PCQ]  “ *Felt comfortable and x settled me down quickly*”[Patient 29, PCQ]  “..*Friendly with a good demeanour*”[Patient 49, PCQ] |

| **Theme 3: PAs Providing Continuity of Care** | |
| --- | --- |
| **Subthemes** | **Illustrative Quotes** |
| Confidence in and of PAs | *“Some junior PAs are a bit more hesitant, but not dissimilar to an FY2 really” [*Consultant 4, Int, 10 years’ experience with PAs]  *“Some PAs are new and need a lot of support. There are some who are clearly not suited to the ED environment and obviously not dissimilar to the doctors.* [Consultant 4, Int, 10 years’ experience with PAs]  *“ I have a good rapport with all my consultants in the department. And so that kind of contributes to helping me with my autonomy and seeing and kind of managing patients with little input”* [PA 1; int, 6.5 years’ experience] |
| PAs Providing Support to the Department | “*I love my role, I love it, it’s great. I mean especially my department I think we are very well supporte*d” [PA 2, Int, 3 years’ experience]  “*My educational supervisor is very supportive and easily approachable*” [PA 1, Int, 6.5 years’ experience]  “*I support the department by providing teaching to ANPs, PAs and students including medical students*” [PA 1, Int, 6.5 years’ experience]  “*Over the last 10 years we have got PAs who are well integrated into the department, and I feel like the department couldn’t live without us now*” [PA 4, Int, 10 years’ experience]  “*supporting role, excellent service*” [Patient 4, PCQ] |
| Partnership and Teamwork in the Department | “ *someone who works alongside with a senior physician*”[Patient 40, PCQ]  “*Medically trained, not quite a doctor’s degree…. Can’t make a big decision by themselves, need to discuss with a senior*” [Patient 44, PCQ]  “*My inability to prescribe is a frustrating but is not a barrier for me, as I have built up trust over the years, that I can go up to a doctor saying may you please prescribe me this fluid for this reason. They are happy to look at the patients themselves and they are happy to prescribe the request. The same with ionisating radiation, they are more than happy to do that*”[ PA 3; Int, 8.5 years experience]. |
| **Theme 4: The Future PA and Regulation** | |
| Role combination | *“I may be able to combine some of my prehospital experiences as a paramedic and my physician associate role”* [PA 2; int, 3 years’ experience] |
| Future Steps for the PA role in the ED | “*A few of them could easily progress to becoming physicians and I think that role and progress in their career should be an option to achieve and supported by the ED by the GMC , BMA, etc*” [Middle grade doctor 2, OLS, > 1 year experience working with PAs] |
| Governance | “*Professional registration know the GMC is set to take on the professional registration and it has been in the pipeline for about three years and they still haven’t got a date for that. So that does affect the fact they can’t prescribe and can’t order XRays etc which is frustrating.”* [Consultant 1, Int, 15 years’ experience working with PAs] |
| Future Workforce Potential | *“ For us in the ED we have to work out how to take it to the next level ensuring PAs are well embedded into the team….What is the career progression as a PA, I think this is a question the health systems are yet to answer as to actually how the PAs fit and progress”*  [Consultant 2, Int, 5 years’ experience working with PAs]  *“ I wouldn’t say PAs are much better, I would say they are equivalent with the same training and they shouldn’t be discriminated against for being a PA rather than being a doctor or ACP. But a lot of people are scared of the PA and it’s got to be a doctor or ACP because they are more established in the UK… There always seems to be a fight between ACP role recruitment in our ED vs PA recruitment”*  [Consultant 1, Int, 15 years’ experience working with PAs]  “*we have 100% retention of PAs in our department, and they all seem happy*” [Consultant 2, Int, 5 years' experience working with PAs]  “*The ED can be very stressful and both physically and mentally demanding*” [PA 1; int, 6.5 years’ experience]  “*I do worry that we in the future will eventually have huge retention problems and you then loose the benefit of having PAs; they all will want rotational jobs*” [Consultant 2, Int, 5 years' experience working with PAs] |
